# l-Lactate dehydrogenase from *Cyanidioschyzon merolae* shows high catalytic efficiency for pyruvate reduction and is inhibited by ATP

**DOI:** 10.1007/s11103-024-01495-0

**Published:** 2024-09-10

**Authors:** Mai Yamamoto, Takashi Osanai, Shoki Ito

**Affiliations:** School of Agriculture, Meiji University, 1-1-1, Higashimita, Tama-Ku, Kawasaki, Kanagawa 214-8571 Japan

**Keywords:** l-Lactate dehydrogenase, Lactic fermentation, Catalytic efficiency, *Cyanidioschyzon merolae*

## Abstract

l-Lactate is a commodity chemical used in various fields. Microorganisms have produced l-lactate via lactic fermentation using saccharides derived from crops as carbon sources. Recently, l-lactate production using microalgae, whose carbon source is carbon dioxide, has been spotlighted because the prices of the crops have increased. A red alga *Cyanidioschyzon merolae* produce l-lactate via lactic fermentation under dark anaerobic conditions. The l-lactate titer of *C. merolae* is higher than those of other microalgae but lower than those of heterotrophic bacteria. Therefore, an increase in the l-lactate titer is required in *C. merolae*. l-Lactate dehydrogenase (l-LDH) catalyzes the reduction of pyruvate to l-lactate during lactic fermentation. *C. merolae* possesses five isozymes of l-LDH. The results of previous transcriptome analysis suggested that l-LDHs are the key enzymes in the lactic fermentation of *C. merolae*. However, their biochemical characteristics, such as catalytic efficiency and tolerance for metabolites, have not been revealed. We compared the amino acid sequences of *C. merolae* l-LDHs (*Cm*LDHs) and characterized one of the isozymes, *Cm*LDH1. BLAST analysis revealed that the sequence similarities of *Cm*LDH1 and the other isozymes were above 99%. The catalytic efficiency of *Cm*LDH1 under its optimum conditions was higher than those of l-LDHs of other organisms. ATP decreased the affinity and turnover number of *Cm*LDH1 for NADH. These findings contribute to understanding the characteristics of l-LDHs of microalgae and the regulatory mechanisms of lactic fermentation in *C. merolae*.

## Key message

ATP inhibited *Cyanidioschyzon merolae* l-lactate dehydrogenase showing high catalytic efficiency for pyruvate reduction, possibly contributing to avoiding the overproduction of ATP via lactic fermentation at night.

## Introduction

Lactate/lactic acid is one of the commodity chemicals used for different fields such as foods, cosmetics, and medicines **(**Abdel-Rahman et al. 2013). Lactate has enantiomers, l-lactate and d-lactate. Both enantiomers are required for manufacturing bioplastic derived from lactate, namely polylactide (Tsuji 2005; Tsuji et al. 2006). Industrial lactate production uses lactic fermentation by microorganisms such as lactic acid bacteria, whose carbon sources are saccharides derived from crops (Ghaffar et al. 2014). However, the prices of the crops have increased because the prices are affected by population growth, soaring crude oil prices, and biofuel production (Bilgili et al. 2020). In recent years, when global warming accelerated, metabolite production from carbon dioxide using eukaryotic microalgae and cyanobacteria is spotlighted. Eukaryotic microalgae and cyanobacteria can produce lactate using carbon dioxide fixed via photosynthesis as the sole carbon source, minimizing the costs of carbon sources such as saccharides (Abdel-Rahman et al. 2013).

*Cyanidioschyzon merolae* is a unicellular red alga living in acid hot springs (pH 1–3 and 40–50 °C) and does not possess a cell wall (De Luca et al. 1978). The genome sequences of the nucleus, mitochondria, and chloroplast in *C. merolae* are completely elucidated (Ohta et al. 1998, 2003; Matsuzaki et al. 2004; Nozaki et al. 2007). Previous transcriptome analysis indicated that *C. merolae* performed anaerobic energy conversion, such as lactic fermentation, rather than aerobic respiration at night (Miyagishima et al. 2019). *C. merolae* produces l-lactate under dark anaerobic conditions (Yoshida et al. 2024). Among eukaryotic microalgae and cyanobacteria, a model cyanobacterium *Synechocystis* sp. PCC 6803 and *Euglena gracilis* also produce l-lactate (Angermayr and Hellingwerf 2013; Tomita et al. 2016). In *Synechocystis* sp. PCC 6803, genetic manipulation is necessary to produce l-lactate because wild-type does not produce l-lactate (Angermayr and Hellingwerf 2013). The l-lactate titer (3.2 g/L) and productivity (16.0–19.4 mg/L/h) of *C. merolae* are higher than those of the *Synechocystis* sp. PCC 6803 mutant (1.8 g/L and 2.7 mg/L/h, respectively) (Yoshida et al. 2024; Angermayr and Hellingwerf 2013). l-Lactate production in *Euglena gracilis* is not efficient because its l-lactate titer is occasionally below 10 mg/L (Tomita et al. 2016). Thus, *C. merolae* is a candidate for a host of l-lactate production from carbon dioxide. However, the l-lactate titer and productivity of *C. merolae* are lower than those of heterotrophic bacteria (Abdel-Rahman et al. 2013), and a further increase in the l-lactate titer of *C. merolae* is required.

l-Lactate dehydrogenase (l-LDH; EC 1.1.1.27) catalyzes the final step in lactic fermentation: pyruvate + NADH → l-lactate + NAD^+^. l-LDH is a paralog of malate dehydrogenase (MDH), and their substrate specificities are determined by five amino acid residues (Yin and Kirsch 2007). l-LDHs have been well characterized in bacteria (particularly lactic acid bacteria) and higher plants (Matoba et al. 2014; Gaspar et al. 2007; Jonas et al. 1972; Barman 1969; Dennis and Kaplan 1960; Götz and Schleifer 1975; Yoshida 1965; Oba et al 1977; Betsche 1981). Bacterial l-LDHs are allosteric enzymes, and fructose-1,6-bisphosphate (FBP) is necessary for their catalytic activities. On the other hand, there are non-allosteric l-LDHs in vertebrate cells (Matoba et al. 2014). Some organisms (*Sporolactobacillus inulinus* YBS 1-5, *Bacillus coagulans, Enterococcus faecalis*, *Enterococcus mundtii* 15-1A, *Fusarium granearum*) possess two isozymes of l-LDH (Wu et al. 2019; Sun et al. 2016; Jönsson et al. 2009; Matoba et al. 2014; Chen et al. 2019). On the other hand, *C. merolae* has five isozymes of l-LDH (Matsuzaki et al. 2004; Nozaki et al. 2007; Ohta et al. 1998, 2003). *C. merolae* does not possess other l-lactate-generating enzymes such as lactaldehyde dehydrogenase and malolactic enzyme (KEGG database URL: https://www.kegg.jp/pathway/map=cme00620&keyword=pyruvate). The expression level of a gene encoding l-LDH increases from day to night in *C. merolae* (Miyagishima et al. 2019), suggesting that l-LDH is the key enzyme in lactic fermentation in *C. merolae*. Previous analysis indicated that the amount of l-LDHs in *C. merolae* remains almost the same under photoautotrophic and dark anaerobic conditions (Yoshida et al. 2024)*.* This suggests that the biochemical regulation of *C. merolae* l-LDHs (*Cm*LDHs) by temperature, pH, and effectors enables them to convert pyruvate to l-lactate under dark anaerobic conditions. We presume that understanding the regulation leads to a further increase in the l-lactate titer of *C. merolae.*

In this study, we compared the amino acid sequences of five *Cm*LDHs (*Cm*LDH1–5) and biochemically analyzed one of the isozymes, *Cm*LDH1.

## Materials and methods

### Preparation of a vector used for the expression of *Cm*LDH1 in *Escherichia coli*

The sequence of the gene encoding *Cm*LDH1 (CMA145C) was acquired from the Kyoto Encyclopedia of Genes and Genomes (KEGG) database (https://www.genome.jp/kegg/kegg_ja.html). The sequence was synthesized by Eurofins Genomics Japan (Tokyo, Japan), and the synthesized sequence was introduced into the *Bam*HI*-Xho*I site of vector pGEX6P-1 (G.E. Healthcare Japan, Tokyo, Japan). The vector was transformed into competent cells of *Escherichia coli* BL21 (DE3) (BioDynamics Laboratory Inc., Tokyo, Japan). After the transformation of the *E. coli*, the *E. coli* cells were cultured in an LB medium (2.4 L) at 30 °C with shaking (150 rpm). During the cultivation, the expression of the recombinant *Cm*LDH1 was induced by 5 µM isopropyl β-d-1-thiogalactopyranoside (Wako Chemicals, Osaka, Japan) overnight.

### Affinity purification of a glutathione-S-transferase (GST) -tagged *Cm*LDH1

The *E. coli* cells in 600 mL culture were suspended in 10 mL phosphate-buffered saline/tween (PBS-T) (0.137 M NaCl, 0.27 mM KCl, 8.1 mM Na_2_HPO_4_.12H_2_O, 1.47 mM KH_2_PO_4_, and 0.001% Tween 20). The cells were sonicated twelve times for 15 s at 20% intensity using model VC-750 (EYELA, Tokyo, Japan). After centrifugation at 14,200 × *g* for 15 min at 4 °C, 800 µL of Glutathione Sepharose 4B resin (G.E. Healthcare Japan, Tokyo, Japan) was added to the supernatant. The sample was gently shaken on ice for 60 min. After that, 10 mM MgSO_4_·7H_2_O and 5 mM ATP were added to the sample, and the mixture was shaken for 30 min at 37 °C. The mixture was centrifugated at 5800 × *g* for 2 min at 4 °C to remove the supernatant. The resin was washed with 3 mL PBS-T five times and 700 µL of PBST five times. The GST-*Cm*LDH1 was eluted by 500 µL glutathione-*S*-transferase (GST) elution buffer [50 mM Tris-HCl (pH 9.6) and 10 mM reduced glutathione] five times. Then, the GST-*Cm*LDH1 was concentrated in a Vivaspin 500 MWCO 30000 device (Sartorius, Göttingen, Germany). The concentration of purified GST-*Cm*LDH1 was measured by a Pierce BCA Protein Assay Kit (Thermo Fisher Scientific, Rockford, IL, USA). Sodium dodecyl sulfate–polyacrylamide gel electrophoresis (SDS-PAGE) was conducted using 8% gels, and the gels were stained by QuickBlue stain reagent (BioDynamics Inc., Tokyo, Japan).

### Enzyme assay

The reaction catalyzed by *Cm*LDH1 proceeded in 1 mL assay solution [100 mM sodium acetate (pH 4.0–5.5), Tris-HCl (pH 7.0–8.0), or the phosphate-citrate buffer (pH 4.0–8.0), different concentrations of sodium pyruvate, NADH, and *Cm*LDH1]. After incubating the assay solution without sodium pyruvate and NADH at different temperature for 5 min, sodium pyruvate and NADH was added to the assay solution to initiate the enzymatic reaction. During the reaction, the decrease in the NADH concentration, namely the change of the absorbance at 340 nm, was monitored for 1 min using a Hitachi U-3900H spectrophotometer (Hitachi High-Tech., Tokyo, Japan). The enzymatic activity of 1 unit was defined as the amount of enzyme that converts 1 μmol of substrate per minute. The *V*_max_ (the maximum reaction velocity) and *S*_0.5_ (the substrate concentration at 1/2 *V*_max_) of *Cm*LDH1 were calculated by curve fitting of the hill equation (Dixon and Webb 1979) (below) using the KaleidaGraph ver. 4.5 software.

$$v = V_{{{\text{max}}}} \left[ {\text{S}} \right]^{{n}{\text{H}}} /\left( {\left[ {\text{S}} \right]^{{n}{\text{H}}} + S_{{0.{5}}}^{{n}{\text{H}}} } \right)$$

The *k*_cat_ (turnover number) were calculated from *V*_max_.

### Cultivation of *C. merolae* and measurement of *Cm*LDH activity in the cell extracts

*Cyanidioschyzon merolae* NIES-3377 (from the National Institute for Environmental Studies) was cultivated in 70 mL of Modified Allen’s medium containing 20 mM (NH_4_)_2_SO_4_ (pH 2.5) at 40 °C (Minoda et al. 2004). During the cultivation, the cultures were bubbled with 1% (v/v) CO_2_ in the air under a white light (25 μmol/m^2^/s photons). After 3 days of the cultivation, the cell density (OD_730_) was measured by a Shimadzu UV-2400 spectrophotometer (Shimadzu, Kyoto, Japan). *C. merolae* cells were recultivated for 3 days from OD_730_ = 0.4. Measurement of *Cm*LDH activity in cell extracts of *C. merolae* were performed as described previously (Yoshida et al. 2024). After 3 days of the cultivation, *C. merolae* cells [OD_730_ × culture volume (mL) = 100] were collected by centrifugation at 5800 × *g* for 2 min. The cells were resuspended in 1 mL of PBS-T [0.137 M NaCl, 2.7 mM KCl, 8.1 mM Na_2_HPO_4_⋅12H_2_O, 1.47 mM KH_2_PO_4_, 0.005% (w/v) Tween-20] and sonicated on ice by a model VC-750 sonicator (EYELA, Tokyo, Japan) at 20% intensity for 10 s. The sonication was repeated five times. The mixture was centrifugated at 17,400 × *g* for 5 min at 4 °C. The total protein concentration in the supernatant was measured by a Pierce BCA Protein Assay Kit (Thermo Fisher Scientific, Rockford, IL, USA), and 200 μg of total proteins was used for enzyme assay.

## Results

**Fig. 1:**
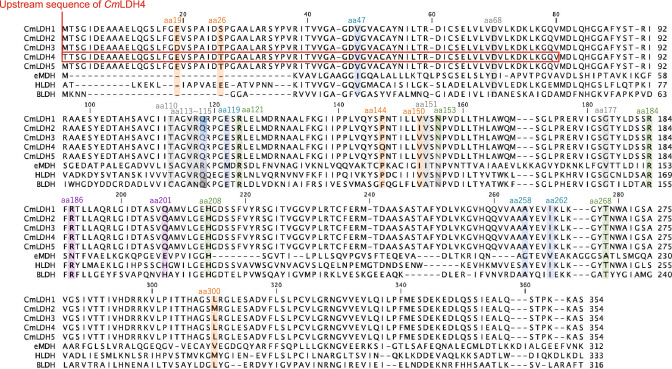
Comparison of amino acid sequences of LDHs and *E. coli* MDH. Amino acid sequences of LDHs and *E. coli* MDH were aligned using CLC Sequence Viewer ver. 8.0. The eMDH, HLDH, and BLDH are *E. coli* MDH, *Homo sapience* LDH, and *Bacillus stearothermophilus* LDH, respectively. The order of amino acid residues of these enzymes is based on that of *Cm*LDH1. The orange squares represent the amino acid residues that differ between *Cm*LDHs (aa19, 26, 144, 150, and 300). The blue squares represent the amino acid residues distinguishing l-LDH and MDH (aa47, 115, 119, 258, and 262) (Yin and Kirsch 2007). The green, gray, and purple squares represent the substrate binding site defined in *H. sapience* LDH (aa121, 153, 184, 208, and 268) (Pineda et al. 2007), NADH binding site defined in *B. stearothermophilus* LDH (aa68, 110, 113, 114, 115, 151, 153 and 177) (Wigley et al. 1992), and FBP binding site defined in *B. stearothermophilus* LDH (aa186 and 201) (Wigley et al. 1992), respectively

We compared the amino acid sequences of five *Cm*LDH isozymes (*Cm*LDH1–5) (Fig. 1). l-LDH is a paralog of MDH, and the five amino acid residues determine l-LDH or MDH (Yin and Kirsch 2007). *Cm*LDH isozymes excluding *Cm*LDH4 possessed the five amino acid residues that are widely conserved in l-LDHs (aa47: Valine, aa115: glutamine, aa119: glutamate, aa258: Alanine, aa262: Isoleucine) (Yin and Kirsch 2007) (Fig. 1). The N-terminal sequence of *Cm*LDH4 was 80 residues shorter than those of the other *Cm*LDHs (Fig. 1). Therefore, *Cm*LDH4 did not possess one of the amino acid residues determining l-LDH or MDH (aa47) (Fig. 1). The BLAST analysis when *Cm*LDH1 was set at a query sequence revealed that the sequence identities and similarities (positives) of *Cm*LDH1 and the other isozymes were 99% and ≥ 99%, respectively (Table 1). *Cm*LDH isozymes excluding *Cm*LDH4 possessed identical amino acid sequences without five amino acids residues (aa19, 26, 144, 150, and 300) (Fig. 1). The five residues were not included in the substrate binding site defined in *Homo sapience* LDH (Pineda et al. 2007) and the NADH binding site defined in *Bacillus stearothermophilus* LDH (Wigley et al. 1992) (Fig. 1). *Cm*LDH4 did not possess one of the amino acids residues composing the NADH binding site defined in *B. stearothermophilus* LDH (aa68) (Fig. 1). An amino acid sequence of *Cm*LDH1 has been used to determine the localization of *Cm*LDHs in the cells as representative *Cm*LDH (Moriyama et al. 2015). Hence, we biochemically characterized *Cm*LDH1 in this study.

**Table 1:** Result of the BLAST analysis of *Cm*LDHs

Entry	Score	E value	Identities	Positives	Gaps
CYME_CMA145C (*Cm*LDH1)	720 bits (1859)	0.0	354/354 (100%)	354/354 (100%)	0/354 (0%)
CYME_CMK006C (*Cm*LDH5)	719 bits (1857)	0.0	353/354 (99%)	354/354 (100%)	0/354 (0%)
CYME_CMC188C (*Cm*LDH2)	719 bits (1856)	0.0	352/354 (99%)	354/354 (100%)	0/354 (0%)
CYME_CMI306C (*Cm*LDH3)	718 bits (1854)	0.0	352/354 (99%)	354/354 (100%)	0/354 (0%)
CYME_CMJ002C (*Cm*LDH4)	563 bits (1452)	0.0	275/276 (99%)	275/276 (99%)	0/276 (0%)

An amino acid sequence of *Cm*LDH1 was used as a query sequence. The BLAST search was performed in the Kyoto Encyclopedia of Genes and Genomes database (https://www.genome.jp/kegg/genome.html)

Identities exhibit the ratio of identical amino acid residues. Positives exhibit the ratio of amino acid residues whose chemical characteristics are similar to amino acid residues in a query sequence

**Fig. 2:**
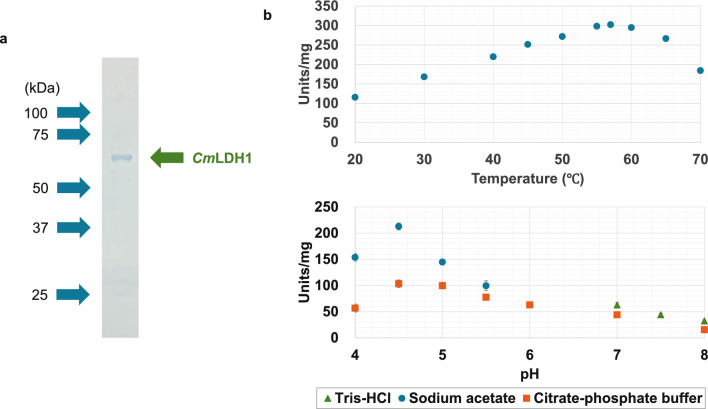
Temperature and pH dependence of *Cm*LDH1 activity. **a** Result of SDS-PAGE after purification of *Cm*LDH1. **b** Effects of temperature (top) and pH (bottom) on *Cm*LDH1 activity. Regarding the measurement of temperature dependence of *Cm*LDH1 activity, pH was fixed at pH 4.5. Regarding the measurement of pH dependence of *Cm*LDH1 activity, the temperature was fixed at 57 °C. The sodium pyruvate and NADH concentrations were 1 mM and 0.15 mM, respectively. The amount of *Cm*LDH1 was 3 pmol. Data exhibit average ± standard deviation obtained from three independent experiments

**Fig. 3:**
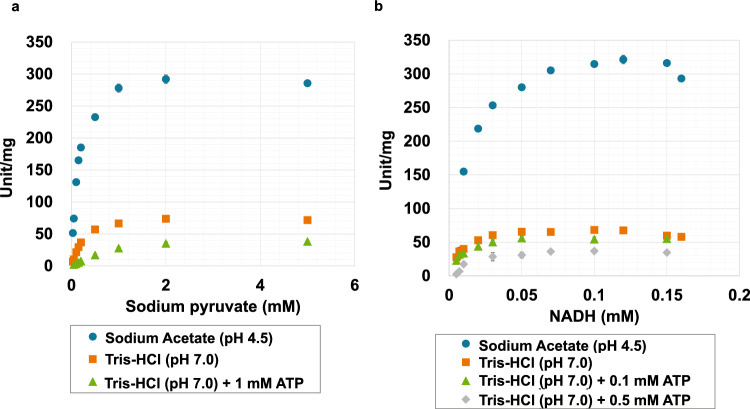
Saturation curves of *Cm*LDH1 for pyruvate and NADH. **a** Saturation curves of *Cm*LDH1 for pyruvate. The experiments were performed under 57 °C and pH 4.5 or 7.0. The concentration of NADH was 0.15 mM. The amount of *Cm*LDH1 was 3 pmol. **b** Saturation curves of *Cm*LDH1 for NADH. The experiments were performed under 57 °C and pH 4.5 or 7.0. The concentration of sodium pyruvate was 2 mM. The amount of *Cm*LDH1 was 0.5 pmol. All data in Fig. 3 exhibit average ± standard deviation from three independent experiments

**Fig. 4:**
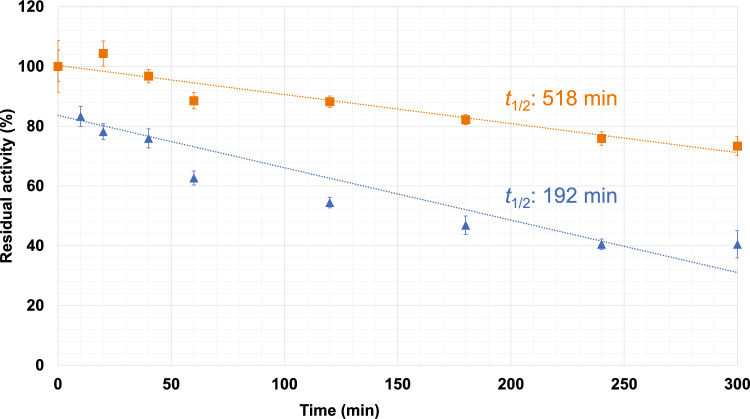
pH stability of *Cm*LDH1. *Cm*LDH1 activities are represented by residual activities, and the activity without incubation at pH 4.5 or 7.0 was 100%. The blue and orange makers indicate residual activities after incubation at pH 4.5 and 7.0, respectively. The temperature was set at 57 °C. The sodium pyruvate and NADH concentrations were 2 mM and 0.15 mM, respectively. The amount of *Cm*LDH1 was 3 pmol. The *t*_1/2_ (time where the residual activity was 50%) was calculated by a linear equation obtained from all the values. Data exhibit average ± standard deviation obtained from three independent experiments

We purified and biochemically characterized a glutathione-S-transferase (GST)-tagged *Cm*LDH1. The single band was localized between 75 and 50 kDa in the SDS-PAGE after purification of *Cm*LDH1 (Fig. 2a). The position of the single band corresponded to the molecular weight of GST-*Cm*LDH1 (63.9 kDa) (Fig. 2a). The purified *Cm*LDH1 exhibited the highest activity under 57 °C and pH 4.5 (Fig. 2b). The *Cm*LDH1 activity on different concentrations of pyruvate and NADH were measured for calculation of kinetic parameters of *Cm*LDH1 under 57 °C and pH 4.5 (Fig. 3). The *S*_0.5_ (the substrate concentration at 1/2 *V*_max_), *k*_cat_ (turnover number), and *k*_cat_/*S*_0.5_ (catalytic efficiency) of *Cm*LDH1 for pyruvate were 0.13 mM, 314 s^–1^, and 2461 s^−1^ mM^−1^ under 57 °C and pH 4.5 (Table 2). The *S*_0.5_, *k*_cat_, and *k*_cat_/*S*_0.5_ of *Cm*LDH1 for NADH were 0.011 mM, 324 s^−1^, and 29,473 s^−1^ mM^−1^ under 57 °C and pH 4.5 (Table 2). The pH of cytosol in *C. merolae* is pH 6.35 to 7.1 (Zenvirth et al. 1985). We also measured the *Cm*LDH1 activity on different concentrations of pyruvate and NADH under 57 °C and pH 7.0 (Fig. 3). The *S*_0.5_, *k*_cat_, and *k*_cat_/*S*_0.5_ of *Cm*LDH1 for pyruvate were 0.20 mM, 79 s^−1^, and 387 s^−1^ mM^−1^ under 57 °C and pH 7.0 (Table 2). The *S*_0.5_, *k*_cat_, and *k*_cat_/*S*_0.5_ of *Cm*LDH1 for NADH were 0.0064 mM, 65 s^−1^, and 10,213 s^−1^ mM^−1^ under 57 °C and pH 7.0 (Table 2). *Cm*LDH1 activity linearly decreased depending on incubation time at pH 4.5 and 7.0 (Fig. 4). The *t*_1/2_ (time where the residual activity was 50%) of *Cm*LDH1 at pH 4.5 and 7.0 was calculated as 192 and 518 min, respectively (Fig. 4).

**Table 2:** Kinetic parameters of *Cm*LDH1

Substrate	pH	Effector	*S*_0.5_ (mM)	*k*_cat_ (s^−1^)	*k*_cat_/*S*_0.5_ (s^−1^ mM^−1^)	*n*_H_
Pyruvate	pH 4.5	None	0.13 ± 0.005	314 ± 3	2461 ± 75	1.10 ± 0.002
	pH 7.0	None	0.20 ± 0.006	79 ± 1	387 ± 6	1.30 ± 0.04
		1 mM ATP	0.61 ± 0.14	43 ± 4**	74 ± 11**	1.42 ± 0.13
NADH	pH 4.5	None	0.011 ± 0.0006	324 ± 6	29,473 ± 1050	1.31 ± 0.05
	pH 7.0	None	0.0064 ± 0.0003	65 ± 2	10,213 ± 748	1.48 ± 0.21
		0.1 mM ATP	0.0069 ± 0.0014	58 ± 1*	8636 ± 1772	1.29 ± 0.23
		0.5 mM ATP	0.013 ± 0.002*	38 ± 2**	2984 ± 386**	2.88 ± 1.82

The measurement conditions were summarized in the legend of Fig. 3. Data exhibit average ± standard deviation obtained from three independent experiments

Kinetic parameters for NADH in the presence of > 0.5 mM ATP cannot be measured because of low activity

*S*_0.5_ the substrate concentration at 1/2 *V*_max_, *k*_cat_ turnover number, *k*_cat_/*S*_0.5_ catalytic efficiency, *n*_H_ Hill coefficient

Asterisks exhibit significant differences between kinetic parameters in the presence and absence of ATP (Welch’s *t*-test: *P < 0.05, **P < 0.005)

**Fig. 5:**
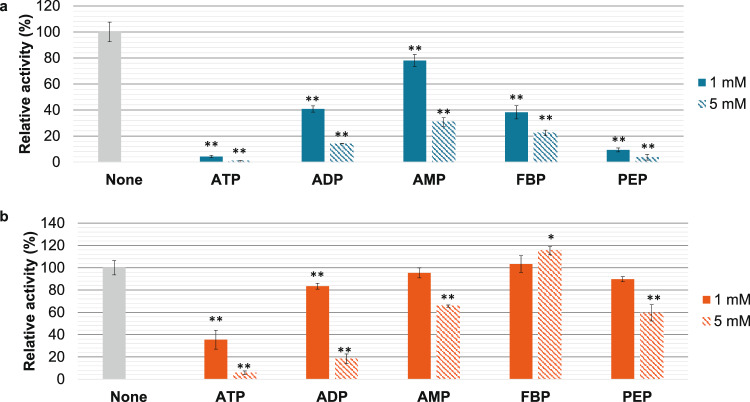
Effects of metabolites on *Cm*LDH1 activity. **a** *Cm*LDH1 activities in the presence of different metabolites under 57 °C and pH 4.5. The concentration of sodium pyruvate was 0.13 mM (*S*_0.5_ at pH 4.5). The concentration of NADH was 0.05 mM because the absorbance change in the presence of inhibitors was not detected when the concentrations of both substrates were *S*_0.5_. The amount of *Cm*LDH1 was 1 pmol. **b** *Cm*LDH1 activities in the presence of different metabolites under 57 °C and pH 7.0. The sodium pyruvate and NADH concentrations were 0.20 mM (*S*_0.5_ at pH 7.0) and 0.05 mM, respectively. The amount of *Cm*LDH1 was 1 pmol. *Cm*LDH1 activity in Fig. 5 was represented by relative activity when the activity in the absence of metabolites was 100%. All data in Fig. 5 exhibit average ± standard deviation from three independent experiments. Asterisks exhibit significant differences between *Cm*LDH1 activities in the presence and absence of metabolites (Welch’s *t*-test: *P < 0.05, **P < 0.005). All metabolites used in this experiment as effectors are sodium salt. *FBP*: Fructose-1,6-bisphosphate, *PEP* Phosphoenolpyruvate

**Fig. 6:**
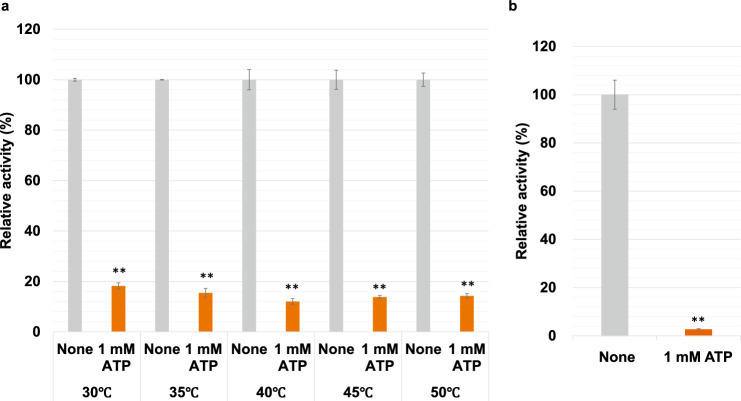
Effect of ATP on *Cm*LDH activities.** a** *Cm*LDH1 activities in the presence of 1 mM ATP at different temperatures. The pH was fixed at pH 7.0. The sodium pyruvate and NADH concentrations were 0.20 mM and 0.05 mM, respectively. The amount of *Cm*LDH1 was 1 pmol. **b** *Cm*LDH activities in cell extracts of *C. merolae* in the presence and absence of 1 mM ATP. The sodium pyruvate and NADH concentrations were 0.20 mM and 0.05 mM, respectively. The amount of total proteins was 200 μg. *Cm*LDH activity in Fig. 6 was represented by relative activity when the activity without ATP was 100%. All data in Fig. 6 exhibit average ± standard deviation obtained from three independent experiments. Asterisks exhibit significant differences between *Cm*LDH activities in the presence and absence of ATP (Welch’s *t*-test: **P < 0.005)

We examined the effect of the five metabolites, effectors of l-LDHs from other organisms, on *Cm*LDH1 (Fig. 5) (Oba et al. 1977; Betsche 1981; Götz and Schleifer 1975; Gaspar et al. 2007; Feldman-Salit et al. 2013; Matoba et al. 2014; Steinbüchel and Schlegel 1983; Davies and Davies 1972). Under 57 °C and pH 4.5, the five metabolites decreased *Cm*LDH1 activity (Fig. 5a). Under 57 °C and pH 7.0, ATP and ADP (particularly ATP) decreased *Cm*LDH1 activity (Fig. 5b). ATP also decreased *Cm*LDH1 activity under 30–50 °C and *Cm*LDH activity in cell extracts of *C. merolae* (Fig. 6). ATP increased the *S*_0.5_ of *Cm*LDH1 for NADH and decreased the *k*_cat_ and *k*_cat_/*S*_0.5_ of *Cm*LDH1 for pyruvate and NADH (Table 2). Under 57 °C and pH 7.0, *Cm*LDH1 activity did not change and decreased in the presence of 1 mM and 5 mM AMP, respectively (Fig. 5b). Under 57 °C and pH 7.0, *Cm*LDH1 activity did not change and increased in the presence of 1 mM and 5 mM FBP, respectively (Fig. 5b). Under 57 °C and pH 7.0, *Cm*LDH1 activity did not change and decreased in the presence of 1 mM and 5 mM phosphoenolpyruvate (PEP), respectively (Fig. 5b).

## Discussion

In this study, we compared the amino acid sequences of five *Cm*LDH isozymes and examined the biochemical properties of *Cm*LDH1, such as catalytic efficiency and tolerance to effectors.

*Cm*LDHs excluding *Cm*LDH4 possessed almost identical amino acid sequences (Fig. 1 and Table 1). Although the N-terminal sequence of *Cm*LDH4 was shorter than those of the other *Cm*LDHs, the upstream sequence of *Cm*LDH4 was similar to the N-terminal sequence of the other *Cm*LDHs (Fig. 1). In Cyanidiophyceae, including *C. merolae*, gene duplications are observed in subtelomeric regions, and the composition of the duplicated genes varies depending on the lineages (Cho et al. 2023). In *C. merolae* genome, all genes encoding *Cm*LDHs are located in the subtelomeric regions (Nozaki et al. 2007). These results suggest that genes encoding *Cm*LDHs were generated by gene duplication in the subtelomeres. Among *Cm*LDHs, only *Cm*LDH4 did not possess amino acid residues equivalent to positions 47 and 68 of *Cm*LDH1 (Fig. 1), suggesting that *Cm*LDH4 cannot catalyze pyruvate reduction.

The catalytic efficiency of *Cm*LDH1 for both substrates at pH 4.5 was higher than those of l-LDHs from other organisms (4 species) (Table 3). The catalytic efficiency of *Cm*LDH1 for pyruvate at pH 7.0 was higher than those of l-LDHs from *Cryptosporidium parvum*, *Limosilactobacillus fermentum*, and *Sporolactobacillus inulinus* and similar to that of *Enterococcus Mundtii* (pH 7.5, 3 mM FBP) (Table 3). The catalytic efficiency of *Cm*LDH1 for NADH at pH 7.0 was higher than those of l-LDHs from *Cryptosporidium parvum* and *Limosilactobacillus fermentum* and similar to that of *Enterococcus Mundtii* (pH 7.5, 3 mM FBP) (Table 3). These comparisons suggest that *Cm*LDH1 is a high-activity l-LDH. Although absolute concentrations (molar concentrations) of pyruvate and NADH in *C. merolae* have been not reported, those of yeast have been reported as those of unicellular eukaryotes (pyruvate: 9.4 mM, NADH: 0.11 mM) (Park et al. 2016). These concentrations of pyruvate and NADH were markedly higher than the *S*_0.5_ of *Cm*LDH1 (pyruvate: 0.13–0.20 mM, NADH: 0.0064–0.011 mM) (Table 2). This result suggests that *Cm*LDH1 shows high activity similar to *V*_max_ in the cells. Absolute quantification of intracellular metabolites of *C. merolae* is also necessary to determine the *Cm*LDH1 activity in the cells accurately in the future. Previous microarray analysis revealed that the expression levels of genes encoding *Cm*LDH and glycolysis enzymes rather than the tricarboxylic acid cycle enzymes increase at night (Miyagishima et al. 2019), suggesting that lactic fermentation is one of the main energy conversions at night in *C. merolae*. The high catalytic activity of *Cm*LDH1 might enable *C. merolae* to perform efficient lactate fermentation at night. The stability of *Cm*LDH1 was higher at pH 7.0 than at pH 4.5 (Fig. 4). Unlike l-lactate production at neutral pH in *C. merolae*, that at acidic pH leads to a decrease in intracellular pH and reaches a plateau at an early period (Yoshida et al. 2024). This might be due to the low stability of *Cm*LDHs at acidic pH.

**Table 3:** The catalytic efficiencies of l-LDHs from various organisms

Organisms	Pyruvate (s^−1^ mM^−1^)	NADH (s^−1^ mM^−1^)	Condition	References
*Cryptosporidium parvum*	0.0105	0.1235	25 °C, pH 5.5	Cook et al. 2015
*Limosilactobacillus fermentum*	5.05	521.9	25 °C, pH 6.0	Lu et al. 2018
*Sporolactobacillus inulinus*	1.4	–	45 °C, pH 7.0	Wu et al. 2019
*Enterococcus Mundtii*	1700	9000	37 °C, pH 5.5, 3 mM FBP	Matoba et al. 2014
*Enterococcus Mundtii*	390	13,000	37 °C, pH 7.5, 3 mM FBP	Matoba et al. 2014
*Cyanidioschyzon merolae*	2461	29,473	57 °C, pH 4.5	This study
*Cyanidioschyzon merolae*	387	10,213	57 °C, pH 7.0	This study

*Cm*LDH1 activity was inhibited by ATP, ADP, and AMP (particularly ATP) in vitro (Fig. 5). These metabolites inhibit l-LDHs from sweet potato roots, *Lactuca sativa L*, and *Staphylococcus epidermidis* (Oba et al. 1977; Betsche 1981; Götz and Schleifer 1975). In *C. merolae*, the concentration of ATP is similar to that of ADP and higher than that of AMP (Miyagishima et al. 2019). Also, the absolute concentration of ATP in yeast (1.9 mM) (Park et al. 2016) is higher than the ATP concentration where ATP inhibited both *Cm*LDHs in cell extracts of *C. merolae* and purified *Cm*LDH1 (1 mM) (Figs. 5 and 6). These results suggest that among the adenine nucleotides, ATP mainly acts as an inhibitor of *Cm*LDH1 in vivo. In *L. sativa* LDH, ATP decreases the affinity for NADH and acts as a competitive inhibitor for NADH (Betsche 1981). In *Cm*LDH1, ATP decreased not only the affinity but also the *k*_cat_ for NADH (Table 2). This suggests that ATP acts as a mixed inhibitor for NADH and does not bind to the NADH binding site in *Cm*LDH1 (Fig. 1). *C. merolae* keeps the adenylate energy charge (balance of adenine nucleotides) almost constant throughout the day/night cycle (Miyagishima et al. 2019). Therefore, we presume that ATP generated via lactic fermentation strongly inhibits *Cm*LDHs to avoid the overproduction of ATP at night.

*Cm*LDH1 activity was affected by FBP and PEP in vitro (Fig. 5). FBP inhibited and slightly activated *Cm*LDH1 activity at pH 4.5 and 7.0, respectively (Fig. 5). The pH of cytosol in *C. merolae* is neutral pH (Zenvirth et al. 1985), suggesting that FBP activates *Cm*LDH activity in vivo. The activation of l-LDHs by FBP has been confirmed in bacteria (*Lactococcus lactis*, *Lactobacillus plantarum*, *Streptococcus pyogenes*, *Enterococcus faecalis*, *Enterococcus mundtii*, *B. stearothermophilus*) (Gaspar et al. 2007; Feldman-Salit et al. 2013; Matoba et al. 2014; Flores and Ellington 2005). The activities of l-LDHs from *L. lactis*, *L. plantarum*, *S. pyogenes*, and *E. faecalis* increase 1000, 1.05, 83, and 7.8-fold in the presence of 3 mM FBP (Gaspar et al. 2007; Feldman-Salit et al. 2013). *B. stearothermophilus* LDH activity increases 15-fold in the presence of 5 mM FBP (Flores and Ellington 2005). Although *Cm*LDH1 activity increased 1.2-fold in the presence of 5 mM FBP (Fig. 5b), the absolute concentration of FBP in yeast (4 mM) is below 5 mM (Park et al. 2016). These results suggest that FBP is not essential for the catalytic activity of *Cm*LDH1. *Cm*LDHs did not possess histidine at position 201 composing the FBP binding site defined in *B. stearothermophilus* LDH (Fig. 1). This might be why *Cm*LDH1 activity hardly depended on FBP. *Cm*LDH1 activity did not change and decreased in the presence of 1 mM and 5 mM PEP at pH 7.0, respectively (Fig. 5b). The inhibition of l-LDHs by PEP has been confirmed in *Cupriavidus necator*, *Ipomoea batatas*, and *Solanum tuberosum* (Steinbüchel and Schlegel 1983; Oba et al. 1977; Davies and Davies 1972). The absolute concentration of PEP in yeast (0.029 mM) is below 1 mM (Park et al. 2016), suggesting that PEP hardly affects *Cm*LDH1 activity in vivo.

This study revealed the biochemical properties of *Cm*LDH1. Our findings contribute to understanding the biochemical characteristics of l-LDHs in microalgae and the regulatory mechanism of lactic fermentation in *C. merolae*. *Cm*LDH1 was inhibited by ATP (Figs. 5 and 6). Therefore, the relief of the inhibition by novel culture methods and genetic manipulation of *C. merolae* might lead to an increase in l-lactate production of *C. merolae*.

## Data Availability

Not applicable.
